# A direct comparison of classical oral Navelbine vs metronomic Navelbine in metastatic breast cancer: results from the Danish Breast Cancer Group’s (DBCG) NAME-trial

**DOI:** 10.1007/s10549-025-07777-5

**Published:** 2025-07-11

**Authors:** Anne Sofie Brems-Eskildsen, Julia Kenholm, Annette Torbøl Brixen, Jeanette Dupont Rønlev, Lars Stenbygaard, Hella Danø, Mie Grunnet, Erik Hugger Jakobsen, Jeppe Neimann, Sven Tyge Langkjer, Jürgen Geisler

**Affiliations:** 1 Department of Oncology, University Hospital Aarhus, Aarhus, Denmark; 2 Department of Oncology, Regional Hospital West Jutland, Goedstrup, Denmark; 3 Department of Oncology, Herlev Hospital, Herlev, Denmark; 4 Department of Oncology, Soenderborg Hospital, Soenderborg, Denmark; 5 Department of Oncology, University Hospital Odense, Odense, Denmark; 6 Department of Oncology, University Hospital Aalborg, Aalborg, Denmark; 7 Department of Oncology, North Zealand Hospital, Hillerod, Denmark; 8 Palliative Unit, AHH, Region H, Hvidovre, Denmark; 9 Department of Oncology, Hospital Lillebaelt, Vejle, Denmark; 10 Department of Oncology, Akershus University Hospital, Norway & Institute of Clinical Medicine, Faculty of Medicine, University of Oslo, Oslo, Norway

**Keywords:** Breast cancer, Metronomic chemotherapy, Metastatic disease, Vinorelbine, Randomized trial

## Abstract

**Purpose:**

The metronomic principle of chemotherapy for malignancies, using frequent small doses, has been suggested to show superior efficacy compared with classical administration. Thus, we aimed at investigating whether treatment with Navelbine, according to the metronomic drug schedule, was superior to conventional oral treatment in terms of clinical efficacy and safety. EUDRACT no: 2016-002165-63.

**Methods:**

The NAME-trial was an open label, randomized, multicenter phase II study. We included 163 patients with metastatic breast cancer in Denmark between 2017 and 2022. All participants were randomized between standard treatment in arm A with classical per oral Vinorelbine day 1 and day 8, every three weeks, or in arm B metronomic treatment with per oral Vinorelbine given as daily doses.

**Results:**

The distribution of patients was well balanced between the two treatment arms. The median age was 68–69 years in both arms, with a good performance status at study entry. We found a median progression-free survival (PFS) in arm A of 3.9 months and a median PFS in arm B of 2.3 months (P = 0.236). The median overall survival (OS) was 16.6 months in arm A and 15.1 months in arm B (P = 0.355). The evaluation of the adverse events showed that both regimes were well tolerated without significant differences.

**Conclusion:**

Our overall evaluation of the NAME-trial results showed that metronomic oral Navelbine is not superior to the standard treatment with Vinorelbine and without any significant differences concerning side effects.

**Supplementary Information:**

The online version contains supplementary material available at 10.1007/s10549-025-07777-5.

## Introduction

Breast cancer is currently the most common cancer among women in the world. In Denmark alone, around 4500 new cases are diagnosed every year. Although several new treatment strategies like CDK4/6 inhibitors and multiple antibody–drug conjugates, to mention some, have been established during the last decade, metastatic breast cancer is still considered incurable with currently available treatment [1–3]. Thus, the goal for treatment of patients with metastatic breast cancer is to relieve symptoms and prolong survival with the best possible quality of life.

During the course of metastatic disease, the majority of metastatic breast cancer patients will at some point be exposed to chemotherapy regimens, but only a few cytotoxic regimens have actually shown a clear survival benefit. Therefore, palliation needs to be balanced against survival and toxicity. In patients, with estrogen receptor (ER)-positive and human epidermal growth factor receptor 2 (HER2)-negative metastatic disease, the choice of treatment depends on multiple factors, including prior treatment, toxicity, performance status (PS), comorbidity, and patient preference.

Interestingly, the optimal sequence of the various regimens is still not completely clarified [3]. In recent years, there has been increased interest in more patient-friendly oral treatments, avoiding intravenous infusions administered at hospitals. Besides capecitabine, oral Navelbine is one of the most widely used oral cytotoxic drugs in metastatic breast cancer [4, 5]. The active drug, vinorelbine, belongs to the group of vinca alkaloids, blocking cell division in G2/M of the cell cycle by inhibiting the assembly of microtubule that is necessary for cell division.

One strategy to obtain chemotherapy effects with possibly reduced side effects has been the so-called “metronomic chemotherapy” [6–9]. Metronomic chemotherapy involves frequent, often daily small doses of the chemotherapeutic drugs that are administered continuously over longer periods of time without major treatment-free periods. The rationale of this concept is not limited to the mode of administration, dose, and schedule. It has been suggested that metronomic chemotherapy may influence tumor vascular endothelial cells and therefore tumor angiogenesis [10, 11] as well as being beneficial for the innate immune system [12, 13]. These potential additional modes of action are based on preclinical studies, as well as clinical observations, suggesting that the administration of small, repeated doses of chemotherapy may have additional beneficial effects [14]. While some investigators have postulated that small but frequently administered doses of a particular drug may lead to a higher dose intensity without corresponding side effects [15], it has not been validated that metronomic cancer therapy is superior to conventional treatment in humans. As a consequence, the present randomized phase II study, called the NAME-trial, was designed to clarify whether treatment with metronomic Navelbine is at least as effective to obtain disease control rate measured by differences in clinical benefit rate and as safe and well tolerated as classical treatment. We hypothesize that it potentially might be superior to conventional treatment with Navelbine in terms of clinical efficacy. The reason for choosing the treatment dose of 30 mg/daily was primarily due to a phase I trial conducted in lung cancer patients [16, 17]. They found the optimal dose of 30 mg, but also with a toxic death in at patient of 77 years. Therefore, we chose a more cautious dose and gave only 30 mg in patients younger than the median aged breast cancer patient (here 65 years old). Patients older than 65 years started on 25 mg and dose escalation was allowed.

## Materials and methods

The NAME-trial was conducted as an open label, randomized, multicenter, phase II study between 2017 and 2022. We included all in all 163 patients with HER-2-negative metastatic breast cancer from nine different centers in Denmark. Both patients with ER-positive and ER-negative breast cancer could be included in the study. The performance status had to be 0–2. Sufficient bone marrow, liver, and kidney function had to be documented at baseline. Patients with asymptomatic CNS metastasis were also accepted in the study. Navelbine treatment could be given as first- to fourth-line chemotherapy for metastatic disease. All patients signed Human Ethics and Consent to Participate. The complete list of inclusion and exclusion criteria is shown in Supplementary Table S1.

**Fig. 1:**
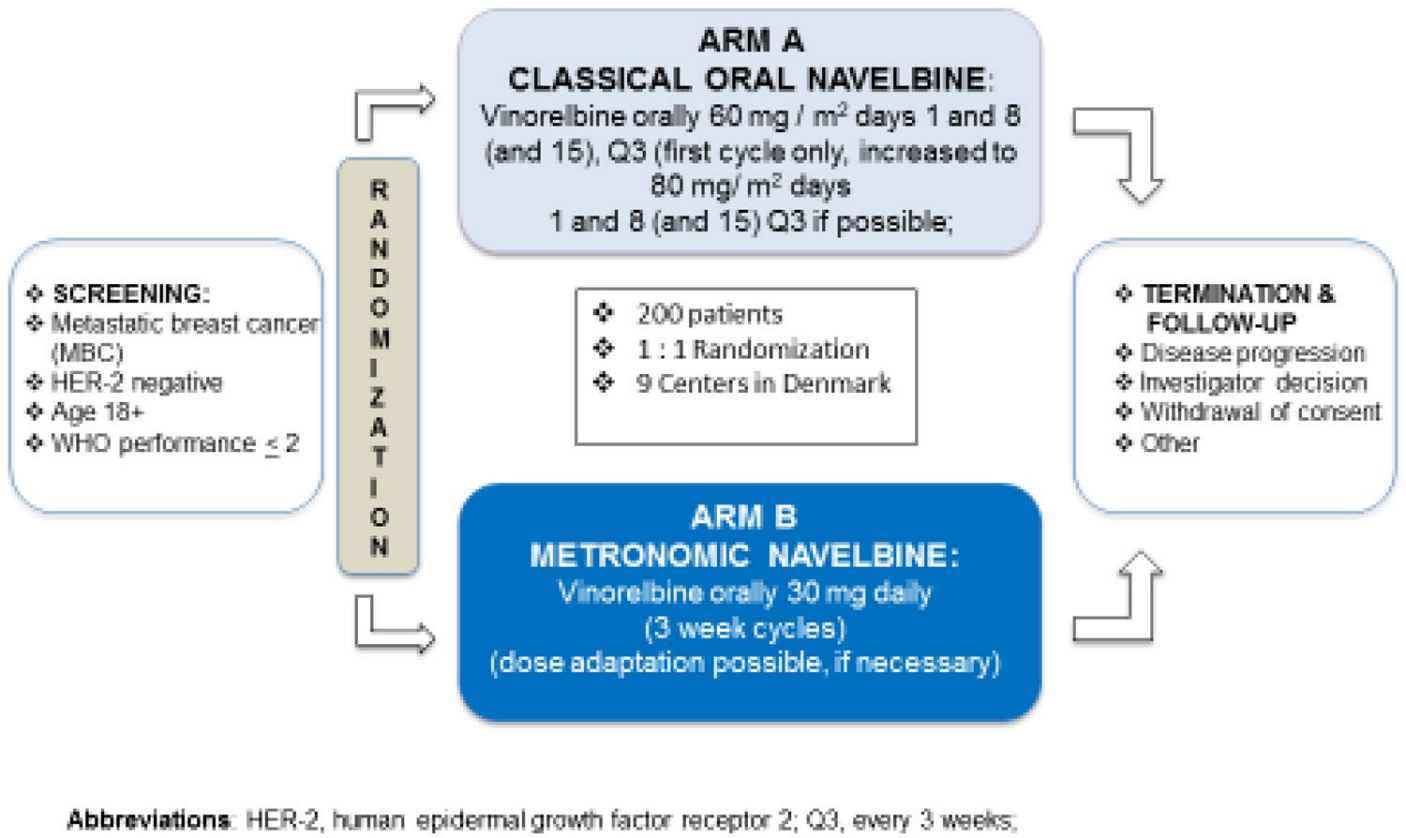
Study design—the NAME-trial

Randomization in the NAME protocol was performed using the electronic case report form (eCRF) Redcap [18, 19] during patient registration and with consent by the user of the participating site. All patients included in the NAME-trial were followed by the REDCAP eCRF designed and adapted especially for this project. The randomization is presented in Fig. 1 and was simply between the standard Arm A and the metronomic experimental Arm B. In Arm A, the patients were treated with “classical” Vinorelbine (Navelbine Oral®): 60 mg/m^2^ day 1 and day 8, every three weeks for the first cycle. Hereafter, 80 mg/m^2^ day 1 and day 8, every three weeks for the following cycle treatment day 15 was optional. For dose reduction options, please see supplementary 2. In Arm B, the patients received “metronomic” Vinorelbine (Navelbine Oral®) meaning 3-week cycles of daily doses of Navelbine. The start dose of Navelbine in arm B was 30 mg once daily and it was possible to reduce the dose to 20 mg once daily (dose level-1) or 30 mg every second day (dose level-2). For more details concerning the dose reduction scheme, please see supplementary S2.

Every patient was treated for at 12 weeks (4 cycles) with Navelbine Oral, and after 3 months of treatment, the tumor responses were evaluated using CT scans and (optional) MR scans, except in cases with obvious early progression of the disease or unacceptable toxicity where the evaluation would be earlier. The RECIST criteria 1.1 [20] were used to evaluate clinical response. Patients continued the study treatment until documented progression of their disease or until severe toxicity occurred. Adverse events were recorded using the Common Terminology Criteria for Adverse Events (version 4.0) [21].

## Statistical methods

For sample size calculation, Simon’s two-stage design for phase II clinical trial was used [22]. We used the null hypothesis H0 for the true disease control rate of 50% along with an alternative hypothesis H1 of 70%, two testing, a one-sided type I error α less than 3.0 and a type II error β less than 10% and we found that 73 evaluable patients per arm should be enrolled. Assuming about 20% of patients will be non-evaluable for several reasons, a total of 100 patients per arm were the goal for this randomized phase II study, leading to an overall sample size of around 200 patients. There were two planned interim analyses in the study. All registered and treated patients were included in the intent-to-treat population. Categorical data are presented in contingency tables with frequencies. Time-dependent survival parameters are described in Kaplan–Meier curves. The study has been designed and executed in accordance with the fifth version of the Declaration of Helsinki, ICH-GCP guidelines, and national laws.

The primary objective was to evaluate clinical benefit rate at three months (CR-PR-SD, SD at three months).

The secondary objective was to evaluate differences in OS and PFS and toxicities in the two groups.

The study was registered and approved in 2016 with EUDRACT number: 2016-002165-63 and approved by the Danish Health Research Ethics Committee with number 1-10-72-305-16. Clinical Trial number: not applicable.

## Results

One hundred and sixty-three patients were included in the study from nine different Departments of Oncology in Denmark. Due to a preplanned interim analysis, the study was prematurely stopped when one hundred and sixty-three out of two hundred planned patients were included, as it was concluded that the metronomic principle would not be superior to the standard treatment with Vinorelbine.

The patients’ characteristics are summarized in Table 1 showing that the study participants were considered acceptably balanced between the two study arms. Of note, 58% of the patients in the standard Arm A and 51% in metronomic Arm B were older than 65 years and the median age was 69 years with a range of 38 to 89 years in Arm A and a median age of 68 years with a range of 40–86 years in Arm B. The distribution of patients concerning their performance status (PS) 0 and 1 was slightly different between the two arms, but there were very few patients in the category PS 2 (please see Table 1 for details). A few more patients in Arm B had ER-negative disease than in Arm A, but the difference was non-significant. The number of patients included in NAME, receiving the treatment as their first chemotherapeutic line treatment, was 23% in Arm A and 27% in Arm B, respectively. Almost all patients with ER-positive disease were treated with anti-hormonal treatment before inclusion in the study and had received 1––4 lines of endocrine treatment prior to chemotherapy. Considering the sites of metastatic disease, the most frequent sites were bone, liver, and lymph nodes. The number of patients with visceral metastases in liver and/or lung was 61% of the patients in Arm A and 49.4% in arm B. There were more patients with liver metastases in arm A than in arm B, but the distribution of patients with any visceral metastasis was well balanced between the two arms (lung and liver metastasis included).

**Table 1:** Patients and tumor characteristics

	Arm A *n* = 86	Arm B *n* = 77	*P* value Chi^2^ test
Age (median) range	69 years 38–89	68 years 40–86	
N = 163	%	%	
Number of patients under and 65 years	32.6	33.8	0.870
Number of patients over 65 years	67.4	66.2	
Gender			
Female	99	99	0.937
Male	1	1	
Performance status (PS)			
0	47	35	0.497
1	43	56	
2	10	6	
Histology type			
IDC	72	86	0.074
ILC	19	12	
Other	9	3	
Primary disseminated			
Yes	31	30	0.83
No	69	70	
Estrogen receptor (ER)			
Negative	10	16	0.330
Positive	89	84	
HER2 receptor			
Negative	98.8	98.7	0.956
Positive	1.2	1.3	
Primary chemotherapy for metastatic disease			
Yes	74	66	0.272
No	26	34	
Number of lines of chemotherapy treatment line before NAME for metastatic disease			
0	26.7	35	
1	44.2	37.7	
2	29	27.3	
Antiestrogen therapy prior to randomization			
Yes	83	74	0.185
No	17	26	
Number of anti-hormone treatments before the NAME-trial for metastatic disease			
0	17.4	26	
1	36	30	
2	32.5	23	
3	10.5	10.4	
4	3.5	10.4	
Metastatic site			
Liver	51.2	33.8	0.03
Lung	18.6	24.7	0.327
Lymph node	26.7	26	0.54
Bone	54.7	49.4	0.55
Mamma	15.1	7.8	0.153
Other	12.8	10.4	0.653
Visceral metastasis (liver and lung)	61.6	49.4	0.158

Most importantly, as seen in Table 2, the objective response rates in the study were 7% in Arm A (standard arm) and 6.5% in Arm B (metronomic arm) (*p* = 0.902), respectively. When including patients with stable disease for more than 180 days, the clinical benefit rates were 19.8% in Arm A and 19.5% in Arm B (*p* = 0.963). We believe that it seems reasonable to include the patients with stable diseases in the response calculation as one of the major goals of palliative treatment is to prevent the disease from evolving.

**Table 2:** Treatment and response in the NAME study

	Arm A	Arm B
	%	%
Reason for exiting		
Progression of disease	81.4	85.7
Death	0	1.3
Adverse events	9.3	7.8
Doctors decision	7.0	3.9
Withdrawal by patient	2.3	1.3
Mortality status		
Death	79	84.4
Alive	20.9	15.6
Cause of death		
Alive or missing	23.3	24.7
Cancer	70.1	72.7
Other	5.8	2.6
Best response		
Complete response (CR)	2.3	3.9
Partial response (PR)	4.7	2.6
Progressive disease (PD)	37	48
Stable disease (SD)	44.2	41.6
Not analyzed	11.6	3.9
Response rate		
Objective response rate (Complete and Partial response)	7	6.5
Clinical benefit rate		
Clinical benefit rate (inclusive stable disease > 180 days/6 months)	19.8	19.5

**Fig. 2:**
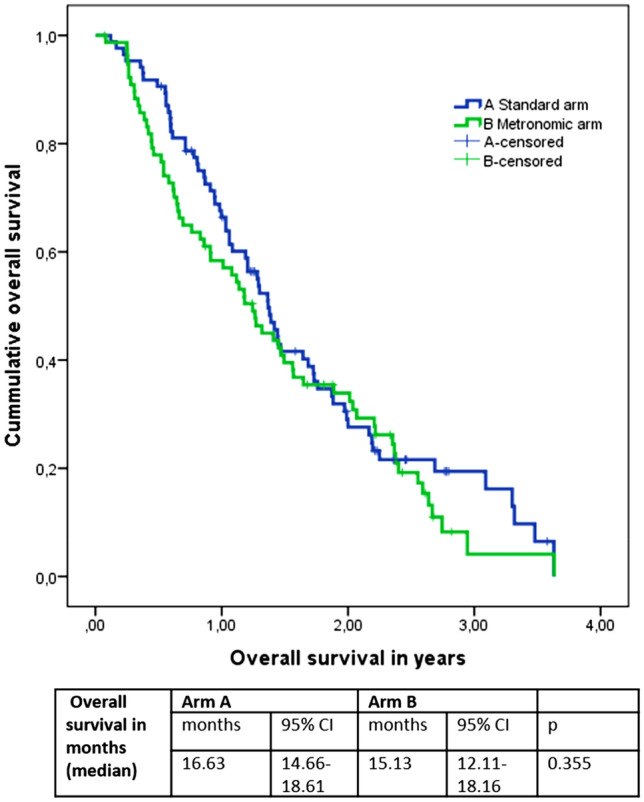
Overall survival in years according to treatment arms

**Fig. 3:**
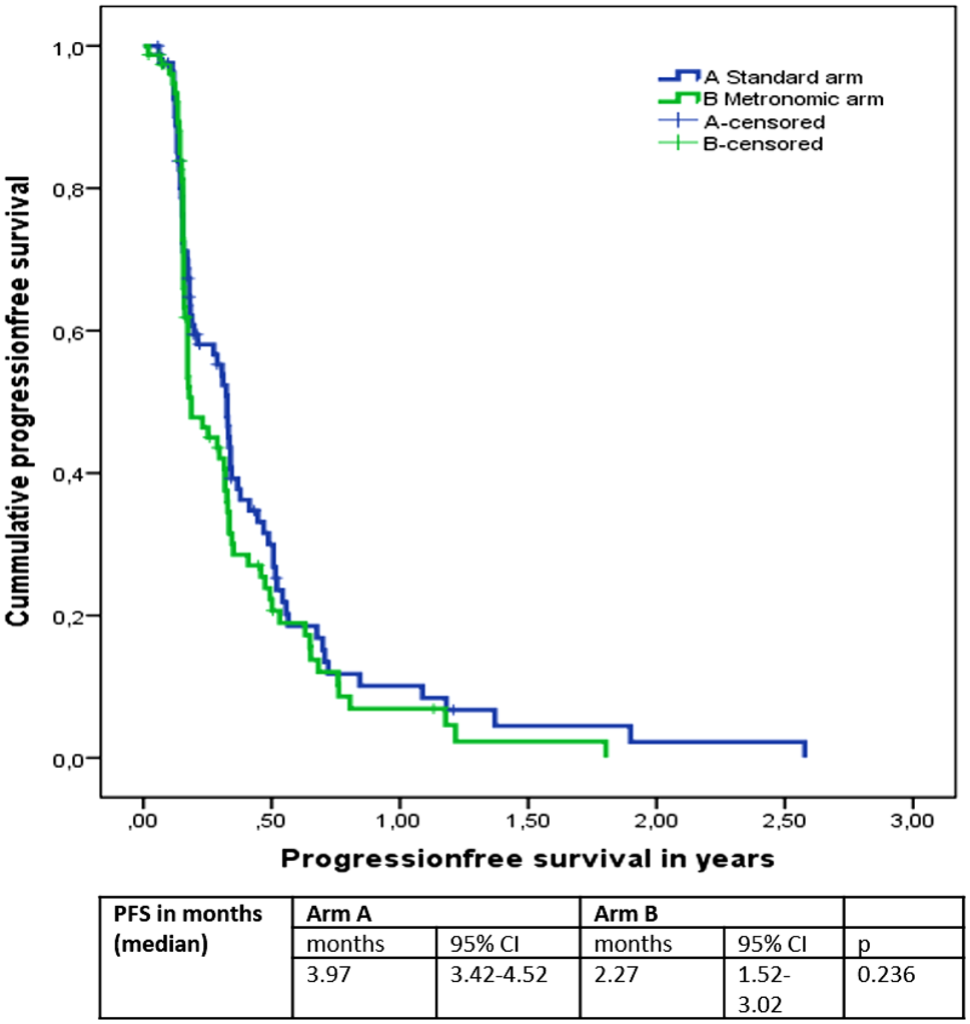
Progression**-**free survival in years according to treatment arms

When looking at the progression-free survival (PFS) and overall survival (OS), we found a median PFS in Arm A of 3.97 months, 95% confidence interval (CI) 3.42–4.52 and a median PFS in Arm B of 2.27 months, 95% CI 1.52–3.02 months (*P* = 0.236). The median OS was 16.63 months (95% CI 14.66–18.61) in Arm A and 15.13 months (95% CI 12.11–18.16) in Arm B (*P* = 0.355). Please see Figs. 2 and 3 for survival presentation in Kaplan–Meier plots.

The median number of cycles in both arms was 3 series with a range in arm A from 1 to 45 series and in arm B with a range from 1 to 31 series. 83% of the patients in arm B and 41% of the patients in arm A were treated per protocol. The low number in arm A was mostly due to no initial dose reduction in the treatment for patients over 65 years. Furthermore, an additional 28% of the patients in arm A and 14% of the patients in arm B were dose reduced later due to side effects. We had 26% interruptions in the treatment in arm A and 31% interruptions in the treatment in arm B. We did not see a difference between the age groups over and below 65 years old regarding efficacy according to different dosing. As the protocol prescribed dose reduction in the population over 65, we cannot conclude whether they needed the dose reduction and benefited from it or not, but we avoided fatal toxicities in the study. In the standard treatment arm, the concern was less and that resulted in higher dose in that population. The following dose reduction and interruption were comparable.

The adverse events found in the study are summarized in Table 3. As can be seen, the regimes were both well tolerated and caused mostly only grade 1 and grade 2 adverse events. The most common hematological adverse event was neutropenia (33.7% in arm A vs 20.8% in arm B) and anemia (16.3 vs 15.6%). Other very common and clinically important side effects were fatigue (65.1% in arm A vs 59.7% in arm B), nausea (43.0 vs 51.9%), and stool changes with both constipation (37.2 vs 28.6%) and diarrhea (46.5 vs 33.8%). The risk of infections was equal and relatively low in both arms (20.9 vs 22.1%) and only 4.7% in arm A and 1.3% in arm B needed treatment for febrile neutropenia (Table 3).

**Table 3:** Adverse events in the NAME study

	Arm A *n* = 86					Arm B *n* = 77				
	Grade adverse events					Grade adverse events				
	Counts				%	Counts				%
	1	2	3–4	Total	Total %	1	2	3–4	Total	Total %
Neutropenia	7	8	14	29	33.7	4	4	8	16	20.8
Leukocytopenia	3	1	1	5	5.8	2	3	1	6	7.8
Thrombocytopenia	1	0	0	1	1.2	3	0	1	4	5.2
Anemia	8	5	1	14	16.3	9	2	1	12	15.6
ALAT/ASAT increased	8	2	0	10	11.6	11	1	1	13	16.8
BASP increased	3	2	1	6	7.0	3	3	0	6	7.8
Creatinine increased	4	2	0	6	7.3	0	2	0	2	2.6
Abdominal pain	4	4	0	8	9.3	4	3	0	7	9.0
Anorexia	12	7	0	19	22.1	10	9	2	21	27.3
Arthralgia	12	5	0	17	19.8	6	10	1	17	22.1
Alopecia	9	7	0	16	18.6	12	5	0	17	22.1
Vision impairment	0	0	0	0	0	0	2	1	3	3.9
Cardiac disease	0	1	2	3	3.5	3	4	2	9	11.7
Confusion	1	0	0	1	1.2	2	1	0	3	3.9
Constipation	23	8	1	32	37.2	12	10	0	22	28.6
Cough	1	2	0	3	3.5	5	2	0	7	9.0
Depression	2	1	0	3	3.5	1	0	0	1	1.3
Diarrhea	26	10	4	40	46.5	18	4	4	26	33.8
Dizziness	5	4	0	9	10.5	3	4	2	9	11.7
Dry eye	2	2	0	4	4.7	1	0	0	1	1.3
Dry mouth	4	0	0	4	4.7	5	0	0	5	6.5
Dry skin	3	0	0	3	3.5	0	0	0	0	0
Dyspepsia	7	3	0	10	10.5	4	2	0	6	7.8
Dyspnea	12	5	3	20	23.3	7	3	2	12	15.6
Edema	3	0	0	3	3.5	2	3	0	5	6.5
Endochrinopathy	1	0	1	2	2.3	0	3	0	3	3.9
Fatigue	29	18	9	56	65.1	25	18	3	46	59.7
Febrile neutropenia	0	0	4	4	4.7	0	0	1	1	1.3
Fever	3	2	0	5	5.8	5	1	0	6	7.8
Headache	3	1	0	4	4.7	2	4	0	6	7.8
Hearing impaired	1	0	0	1	1.1	1	1	0	2	2.6
Hypertension	2	4	7	13	15.1	1	5	3	9	11.7
Infection	2	11	5	18	20.9	0	10	7	17	22.1
Insomnia	5	0	0	5	5.8	1	1	0	2	2.6
Mucositis	13	6	0	19	22.1	10	4	0	14	18.2
Myalgia	9	1	0	10	10.5	11	3	0	14	18.2
Nail changes	2	3	0	5	5.8	0	3	0	3	3.9
Nausea	23	12	2	37	43.0	22	15	3	40	51.9
Neuropathies	16	7	0	23	26.7	15	2	2	19	24.7
Pain	16	12	2	30	34.9	13	19	4	36	46.8
PPE	1	0	0	1	1.2	4	0	0	4	5.2
Tremor	1	1	0	2	2.3	1	1	0	2	2.6
Vomiting	12	5	1	18	20.9	15	1	3	19	24.7

Number of adverse events in the 2 arms. As can be seen, we have very few grade 3–4 adverse events

## Discussion

The clinical responses to systemic chemotherapy in metastatic breast cancer are not satisfying, and many patients experience severe side effects during their cancer treatment. Thus, the search for novel strategies to administer chemotherapy in a more effective and less toxic way is still ongoing. Among several others, the metronomic approach to cancer treatment has been one of the most appealing regimens [23, 24]. The basic idea behind metronomic therapy is to give many small doses of a certain drug to allow a more or less continuous therapy with potentially lower side effect rates and perhaps additional anti-cancer effects like modifications of the tumor vascularization and positive effects on the intra-tumoral immune environment [25–28].

In the NAME-trial presented here, we investigated metronomic chemotherapy in metastatic breast cancer patients. Patients were randomized between the classical way to administer Navelbine (day 1 and 8 every 3 weeks) and the exploratory, metronomic approach with daily doses of Navelbine. After the inclusion and treatment of 163 patients, a planned interim analysis of the clinical outcome made it clear that the metronomic therapy given to all patients in arm B was not superior to the classical treatment in arm A. In fact, we saw a trend toward a shorter progression-free survival in the metronomic arm B (2.27 months) compared to the classical treatment in arm A (3,97 months), although not reaching the level of statistical significance. The objective response rates in both arms were very small but similar (6–7%), while the clinical benefit rate was 19.8 and 19.5%, respectively, when including the patients with stable disease for more than 180 days/6 months.

One might wonder whether the dosage of the daily treatment with Navelbine in the metronomic arm B could explain at least partly our results. The chosen dose in the NAME-trial was 30 mg once daily in the metronomic arm for patients under 65 years of age and due to a concern for bone marrow toxicity 20 mg once daily for patients over 65 years and based on phase I data where daily dose of 20–50 mg were tested on a three-week schedule and one-week treatment break [16, 17]*.* The recommended dose from the phase I trial was 30 mg and the possibility to increase the dose to 40 mg if well tolerated. Since we wanted to test daily dosing without breaks, we chose 30 mg and 25 mg in patients over 65 years. Vinorelbine has been reported to be toxic and that was the reason for the cautious and conservative age cut. The dose for the three-week dosing was based on our experience from another randomized trial (the XeNa-trial) [29]. In the XeNa-trial, patients were randomly assigned to either vinorelbine weekly in combination with capecitabine in standard dose or vinorelbine 3 times weekly in combination with capecitabine in standard dose. No significant difference in effect was found in the XeNa-trial, and the toxicities in both arms were acceptable. We did not see a difference between the age groups over and below 65 years old regarding efficacy according to different dosing. As the protocol prescribed dose reduction in the population over 65, we cannot conclude whether they needed the dose reduction and benefited from it or not, but we avoided fatal toxicities in the study. In the standard treatment arm, the concern was less and that resulted in higher dose in that population. The following dose reduction and interruption were comparable.

Another clinical trial involving metronomic Navelbine that should be mentioned here was the VinoMetro study [30]. This trial was also initiated in 2021 and tested the same daily dose of Navelbine as in the NAME-trial (30 mg/daily) monotherapy but had to be closed prematurely due to a toxic death after inclusion of only 9 patients. Although the patient number was very low, the authors reported a clinical benefit rate (CBR) of 22.2% after approximately 6 months close to the CBR found in the NAME study of 19.5% in the metronomic arm after 6 months. In contrast to the VinoMetro-trial, we did not see any grade 4 and 5 adverse events (AE), but our protocol also had a planned dose reductions in patients of 65 years and above to a standard start dose of minus 1 (20 mg/day).

Finally, the results from the Tempo Breast study [30, 31] have recently been published. Tempo Breast was the first phase 2, randomized clinical trial comparing monotherapy treatment with vinorelbine 50 mg three times weekly (metronomic) to weekly vinorelbine (60 mg/m^2^ increasing to 80 mg/m^2^ in second cycle) as the standard dosing. The authors found a median PFS of 4.0 and 5.6 months, and a median overall survival rate of 22.3 and 26.7 months, respectively, in the metronomic arm and standard arm. The objective response rate was 17.1% in the metronomic arm and 21% in the standard arm, while the disease control rate (defined as CR + PR + SD more than 6 months) was 63.4 and 72.8%, respectively. Interestingly, the previously published Tempo Lung trial [32], had shown a significant difference in clinical responses in favor of the metronomic treatment to lung cancer patients with 50 mg three times weekly.

Metronomic treatment regimens have previously been reported in the large retrospective Victor 6 study [33], this included 584 patients with advanced breast cancer where data were collected from 2011 to 2016. Different chemotherapy regimens were used, among them vinorelbine monotherapy. The authors report impressive response rates of especially vinorelbine monotherapy regimes, where in the first-line chemotherapy setting, overall response rate of 44% and disease control rate of 88% were reported. In the second and third line setting, the overall response rate dropped to 17 and 15% and the disease control rate dropped to approximately 65 and 52%. While these findings are superior to the results found in the present NAME-trial, the reasons for the observed differences are unknown at this stage.

## Conclusion

The NAME study was conducted by the Danish Breast Cancer Group to provide evidence that metronomic chemotherapy with daily dosing of vinorelbine was safe, tolerable, and potentially superior to classical maximal tolerable dosing of chemotherapy using a randomized trial design for optimal design. Several scientific publications had previously suggested the metronomic treatment principle to be clinically effective while less toxic. Unfortunately, we report here no statistical differences between the two treatment arms concerning clinical efficacy. However, both treatment options were all well tolerated with acceptable adverse event profiles and no significant differences considering the risk of myelosuppression or gastrointestinal toxicities. No unexpected signals regarding safety were observed.

In conclusion, metronomic chemotherapy has been suggested to exert certain additional anti-tumor effects, like angiogenic events and positive effects on the tumor immune environment, but in the current clinical study, the NAME-trial, we did not see that these are translated into a superior efficacy in metastatic breast cancer compared to the established classical regimen.

## Supplementary Information

Below is the link to the electronic supplementary material.

Supplementary file1 (DOCX 21 KB)

## Data Availability

Data is provided in the manuscript. Raw data can be provided if needed from the corresponding author.

## Abbreviations

CDK4/6 inhibitors: Cyclin D kinase inhibitors
ER: Estrogen receptor
HER2: Human epidermal growth factor receptor 2
PS: Performance status
CNS: Central nervous system
eCRF: Electronic case report form
PFS: Progression-free survival
OS: Overall survival
CI: Confidence interval
CBR: Clinical benefit rate
AE: Adverse events
CR: Complete response
PR: Partial response
PD: Progressive disease
OR: Objective response rate
SD: Stable disease
