# Supplementary material for: A direct comparison of classical oral Navelbine vs metronomic Navelbine in metastatic breast cancer: results from the Danish Breast Cancer Group’s (DBCG) NAME-trial
Source: Breast Cancer Res Treat. 2025 Jul 11;213(2):237–46. doi: 10.1007/s10549-025-07777-5 (PMC12331846; doi:10.1007/s10549-025-07777-5)
Supplement: Supplementary file 1 — Supplementary file1 (DOCX 21 KB) [file 10549_2025_7777_MOESM1_ESM.docx]

**Supplementary 1: Inclusion and exclusion criteria in the NAME trial.**

**INCLUSION CRITERIA:**

1 Patients with histologically confirmed metastatic breast cancer. The tumor cells should be tested HER2 negative from the primary tumor or by biopsy of metastatic sites (measured with ICH 0-1 + or ICH 2+ with negative FISH, CISH)

2 An expected lifetime of more than 12 weeks.

3 Age over 18 years.

4 WHO performance status <2

5 Patients who, after oral and written information, agree to be included in the study (written consent).

6 Patients that is suitable for first, second, third and fourth line treatment with Navelbine.

7 Patients, with only a solitary lesion, must have cytological or histologically verification, if the lesion represents the only evidence of the disease.

8 Women of childbearing potential must be using a medically accepted method of contraception to avoid pregnancy during the 2 months preceding the start of study treatment, throughout the study period and for up to 3 months after the last dose of study treatment in such a manner that the risk of pregnancy is minimized (see Exclusion criteria's).

9Women of childbearing potential must have a negative serum or urine pregnancy test within 72 hours prior to the start of study treatment;

10 Fertile men must be using an effective method of birth control if their partners are women of childbearing potential throughout the study period and for up to 3 months after the last dose of study treatment;

**EXCLUSION CRITERIA:**

1. Patients that have received Navelbine treatment earlier.

2. Parallel treatment with another anti-cancer treatments for their metastatic disease.

3. Previous treatment with cytostatic within 3 weeks prior to the initiation of the study.

4. Patients with peripheral sensory neuropathy > degree II

5. Malabsorption syndrome or previous surgeries with resection of the stomach or small intestine, whereby absorption of Navelbine may be affected.

6. Difficulty in swallowing tablets.

7. Pregnant or breast-feeding women. There must be a negative pregnancy test (urine test) during the screening period for women of childbearing potential.

8. Women of childbearing potential, who are not using adequate contraception.

The following list of births control methods are considered as adequate:

combined (estrogen and progestogen containing) hormonal contraception associated with inhibition of ovulation

- progestogen-only hormonal contraception associated with inhibition of ovulation:
- intrauterine device (IUD)
- intrauterine hormone-releasing system (IUS)
- bilateral tubal occlusion
- vasectomized partner
- sexual abstinence (if refraining from heterosexual intercourse during the entire period of risk associated with the study treatments. The reliability of sexual abstinence needs to be evaluated in relation to the preferred and usual lifestyle of the subject).
- Adequate contraception must be used during treatment and at least 3 months after last dose of Navelbine. (see Inclusion Criteria)

9. Clinical symptoms of CNS metastasis requiring large doses of steroids.

10. Decreased bone marrow function defined by, neutrophil counts <1.0 x 10^9^ / l and platelets <100 x 10^9^ / l.

11. Hepatic impairment defined by bilirubin > 1.5 x upper normal limit (UNL) and / or ALT > 2.5 x UNL. Renal impairment defined by se-creatinine > 1.5 x UNL or Creatinine clearance <50 ml / min.

12. Other severe medical conditions, including serious heart disease, unstable diabetes, uncontrolled hypercalcemia, clinically active infections or previous organ transplants.

13. Participation in another clinical trial with experimental medication within 30 days prior to registration.

14. Vaccination with Yellow fewer-vaccine or any live virus vaccine. Inactivated annual influenza vaccination is allowed.

15 Patients who needs long-term oxygen treatment.

16 Patients that are allergic to Navelbine, other vincaalkaloids or have known hypersensitivity to any excipient contained in the drug formulation

**Supplementary 2: Treatment plan and dose reduction principle**

**TREATMENT PLAN**

**Arm A**

**Standard treatment with either**

**Vinorelbine (Navelbine Oral^^):** **60 mg/m²** **day 1 and day 8,** every three weeks for the first cycle. Hereafter 80 **mg/m²** **day 1 and day 8,** every three weeks for de following cycles.

**Or standard treatment with:**

**Vinorelbine (Navelbine Oral^^):** **60 mg/m²** **day 1, day 8 and day 15,** every three weeks for the first cycle. Hereafter **80 mg/m²** **day 1, day 8 and day 15** every three weeks for de following cycles.

For dose reduction scheme, see below.

**Patients ≥ 65 years start on dose 60 mg/m2 and the dose is not increased. If Vinorelbine 80 mg/m^2­^ is too toxic it is allowed for the investigator to return to use Vinorelbine 60 mg/m^2­^**

| Standard dose arm A | | | | | | | |
| --- | --- | --- | --- | --- | --- | --- | --- |
| **Vinorelbine 60 mg/m^2­^** | | | | **Vinorelbine 80 mg/m^2­^ PO** | | | |
| **Surface** | **Dose level** | | | **Surface** | **Dose level** | | |
| **m^2^** | **0** | **-1 (75 %)** | **-2/(50%)** | **m^2^** | **0** | **-1 (75 %)** | **-2/(50%)** |
| ≤ 1,54 m² | Vinorelbine  90 mg | Vinorelbine 70 mg | Vinorelbine  40 mg | ≤ 1,54 m² | Vinorelbine 120 mg | Vinorelbine  90 mg | Vinorelbine 60 mg |
|  |  |  |  |  |  |  |  |
| 1,55- 1,71  m^2^ | Vinorelbine  100 mg | Vinorelbine 80 mg | Vinorelbine  50 mg | 1,55-1,71  m² | Vinorelbine 130 mg | Vinorelbine  100 mg | Vinorelbine  60 mg |
|  |  |  |  |  |  |  |  |
| 1,72-1,90 m² | Vinorelbine  110 mg | Vinorelbine 80 mg | Vinorelbine 50 mg | 1,72-1,90 m² | Vinorelbine 140 mg | Vinorelbine 110 mg | Vinorelbine 70 mg |
|  |  |  |  |  |  |  |  |
| > 1,90 m² | Vinorelbine 120 mg | Vinorelbine 90 mg | Vinorelbine 60 mg | > 1,90 m² | Vinorelbine 160 mg | Vinorelbine 120 mg | Vinorelbine 80 mg |
|  |  |  |  |  |  |  |  |

**Arm B**

**Vinorelbine (Navelbine Oral^®^):** 3 week cycles of daily doses of Navelbine is given.

For dose reduction scheme, see below.

| **Standard dose arm B** | | |
| --- | --- | --- |
| **Dose level** | | |
| **0** | **-1** | **-2** |
| **Vinorelbine 30 mg** | **Vinorelbine 20 mg** | **30 mg every other day** |

**Dose adjustment**

Before treatment, normal blood count is needed.

**Hematological toxicity in general**

During the first 2 cycles of therapy a weekly blood count is needed. If the blood count is stable, only blood counts just before next treatment is needed (ex at day -1).

If ANC > 1,0 on day -1 of the planned treatment day and the patient is well, the treatment can be given as planned, otherwise the treatment must be postponed.

**Permanent dose reduction of Vinorelbine to dose level -1 at febrile neutropenia** (temperature > 38,5^o^ C with ANC < 1,0 x 10^9^/l) or nadir of thrombocytes < 25 x 10^9^/l.

If grade 3 toxicity occurs, ANC > 0,5- < 1,0 x 10^9^/l and /or thrombocytes < 50 x 10^9^/l at the planed treatment day the treatment should be postponed until recovery to > 1,0 x 10^9^/l and thrombocytes > 50 x 10^9^/l. Re-initiate Vinorelbine at same dose level. If toxicity recurs at grade 3: temporary dose interruption until recovery to > 1,0 x 10^9^/l and reduce to the next lower dose level.

If grade 4 toxicity occurs, ANC < 0,5 x 10^9^/l and /or thrombocytes < 25 x 10^9^/l at the planed treatment day the treatment should be postponed until recovery to > 1,0 x 10^9^/l.

Re-initiate Vinorelbine at the next lower dose level. If toxicity recurs at grade 4: temporary dose interruption until recovery to > 1,0 x 10^9^/l and reduce to the next lower dose level.

If treatment is postponed more than 4 week the patient must discontinue Vinorelbine

and go of study.

**Neurotoxicity**

In case of neurological symptoms including a distension of the abdomen, which are graded as grade 3 or 4 after CTC, discontinue Vinorelbine until the symptoms decrease to grade 2 or less. The dose is subsequently reduced by 1 dose step and this dose is considered 100% dose for subsequent courses.

Hepatotoxicity

If the bilirubin’s increase during treatment to more than twice the upper normal value (2N) and / or ALAT elevations of more than 3 N (unrelated to liver metastases) discontinue Vinorelbine until the symptoms decrease to grade 1 or less. The dose of Vinorelbine should be reduced to dose level -2 in ARM A and dose level -1 in ARM B in the subsequent treatments. If the toxicity persists for more than 2 weeks, the patient has to go off study.

Other toxicity, apart from hair loss

By any side effect that is classified as grade 3 or 4, a dose reduction of 1 dose step in subsequent cycles of Vinorelbine should be made.
